# Model-Based Fluid-Structure Interaction Approach for Evaluation of Thoracic Endovascular Aortic Repair Endograft Length in Type B Aortic Dissection

**DOI:** 10.3389/fbioe.2022.825015

**Published:** 2022-06-23

**Authors:** Arian Aghilinejad, Heng Wei, Gregory A. Magee, Niema M. Pahlevan

**Affiliations:** ^1^ Department of Aerospace and Mechanical Engineering, University of Southern California, Los Angeles, CA, United States; ^2^ Division of Vascular Surgery and Endovascular Therapy, Department of Surgery, Keck School of Medicine, University of Southern California, Los Angeles, CA, United States; ^3^ Division of Cardiovascular Medicine, Department of Medicine, University of Southern California, Los Angeles, CA, United States

**Keywords:** aortic dissection, endovascular repair, blood pressure, hemodynamics, fluid-structure interaction, medical therapy

## Abstract

Thoracic endovascular aortic repair (TEVAR) is a commonly performed operation for patients with type B aortic dissection (TBAD). The goal of TEVAR is to cover the proximal entry tear between the true lumen (TL) and the false lumen (FL) with an endograft to induce FL thrombosis, allow for aortic healing, and decrease the risk of aortic aneurysm and rupture. While TEVAR has shown promising outcomes, it can also result in devastating complications including stroke, spinal cord ischemia resulting in paralysis, as well as long-term heart failure, so treatment remains controversial. Similarly, the biomechanical impact of aortic endograft implantation and the hemodynamic impact of endograft design parameters such as length are not well-understood. In this study, a fluid-structure interaction (FSI) computational fluid dynamics (CFD) approach was used based on the immersed boundary and Lattice–Boltzmann method to investigate the association between the endograft length and hemodynamic variables inside the TL and FL. The physiological accuracy of the model was evaluated by comparing simulation results with the true pressure waveform measurements taken during a live TEVAR operation for TBAD. The results demonstrate a non-linear trend towards increased FL flow reversal as the endograft length increases but also increased left ventricular pulsatile workload. These findings suggest a medium-length endograft may be optimal by achieving FL flow reversal and thus FL thrombosis, while minimizing the extra load on the left ventricle. These results also verify that a reduction in heart rate with medical therapy contributes favorably to FL flow reversal.

## Introduction

Aortic dissection is a catastrophic life-threatening aortic emergency that can result in aortic rupture, myocardial infarction, pericardial tamponade, stroke, acute kidney failure, bowel ischemia, lower extremity ischemia, and in the long-term congestive heart failure and aortic aneurysms (Prêtre and Von Segesser, 1997; Collins et al., 2004; Januzzi et al., 2004). Acute aortic dissection is a tear in the aortic wall, resulting in high-pressure blood flow through a false passage within the smooth muscle layer of the aorta, creating a false lumen (FL) channel. This FL may flow back into the original aortic flow channel (the true lumen; TL) distally or proximally from the original tear. Anatomically, aortic dissections are categorized into Stanford type A involving the ascending aorta and Stanford type B aortic dissection (TBAD) which occurs in the aortic arch or distally, and usually extend down to the thoracoabdominal aorta (Baliga et al., 2007). While type A dissections typically undergo immediate open repair of the ascending aorta, the thoracoabdominal segment of aorta cannot be repaired at the same time, so patients are typically left with a residual dissection, which is anatomically similarly to a *de novo* type B dissection (Girish et al., 2016; Magee et al., 2019). The first line treatment for TBAD is medical treatment to decrease systolic blood pressure and heart rate which decreases the risk of rupture and progression of disease, but there is growing evidence that early thoracic endovascular aortic repair (TEVAR) may result in improved outcomes to medical management alone (Baliga et al., 2007; Thrumurthy et al., 2011; Magee et al., 2019). TEVAR for TBAD occludes the flow of blood across the proximal aortic tear and shunts it back into the TL. This decompresses the FL, causes thrombosis within the FL, and thereby allows it to heal (Mathlouthi et al., 2021).

By decreasing FL flow, TEVAR thus allows for aortic healing and decreases the risk of subsequent aortic aneurysm and rupture (Thrumurthy et al., 2011; Girish et al., 2016; Yazdani et al., 2017; Yazdani et al., 2018; Yin et al., 2022). Clinical data have found that TBAD patients with complete FL thrombosis have improved outcomes, whereas failure of FL thrombosis, and persistent FL flow is a predictor of adverse outcomes (Williams et al., 1997; Tsai et al., 2007; Thrumurthy et al., 2011). However, flow patterns in TBAD are poorly understood due to the complexity of patient-specific anatomy and physiology as well as the limitations of imaging modalities (Birjiniuk et al., 2020). While TEVAR has shown promising results in the treatment of TBAD patients, the permanent implantation of a prosthetic endograft can cause its own set of problems including spinal cord ischemia with resulting paralysis, stroke, and long-term heart failure. Current endografts have significantly great stiffness and anisotropy compared to the native aorta (Tai et al., 2000). The compliance mismatch between the endograft and the native aorta can lead to a cascade of hemodynamic alterations which affect the aortic wave dynamic and may contribute to subsequent cardiovascular complications such as congestive heart failure (Vlachopoulos et al., 2010; Takami et al., 2012). Deleterious effects of compliance mismatch can even occur proximal to the endograft by affecting delicate hemodynamic balance between the left ventricle (LV) and vascular network which exists in normal physiological condition (Kolh et al., 2000). Therefore, much remains to be understood about the biomechanical consequences of TEVAR for TBAD and what length of endograft is optimal for treatment.

The objective of this study is to evaluate the impact of endograft implantation in TEVAR on the unique fluid dynamics behavior of the pulsatile blood flow in the TL and FL. We used an idealized geometry to focus on the overall behavior of the hemodynamics independent of individual patient anatomy. Due to the extensive endograft-related variability in TEVAR, this study focused primarily on the impact of endograft length. We examined the impact of endograft length on the LV pulsatile workload (as an indicator of global cardiovascular state (Pahlevan and Gharib, 2011a; Pahlevan and Gharib, 2013; Aghilinejad et al., 2021a)) and the FL flow reversal (as a predictor for FL thrombosis (Birjiniuk et al., 2017; Birjiniuk et al., 2019)). While the optimal treatment modality for type B dissection is currently the subject of considerable debate, this study provides insight on the impact of TEVAR on aortic fluid dynamics.

## Materials and Methods

### Physical Problem

**FIGURE 1:**
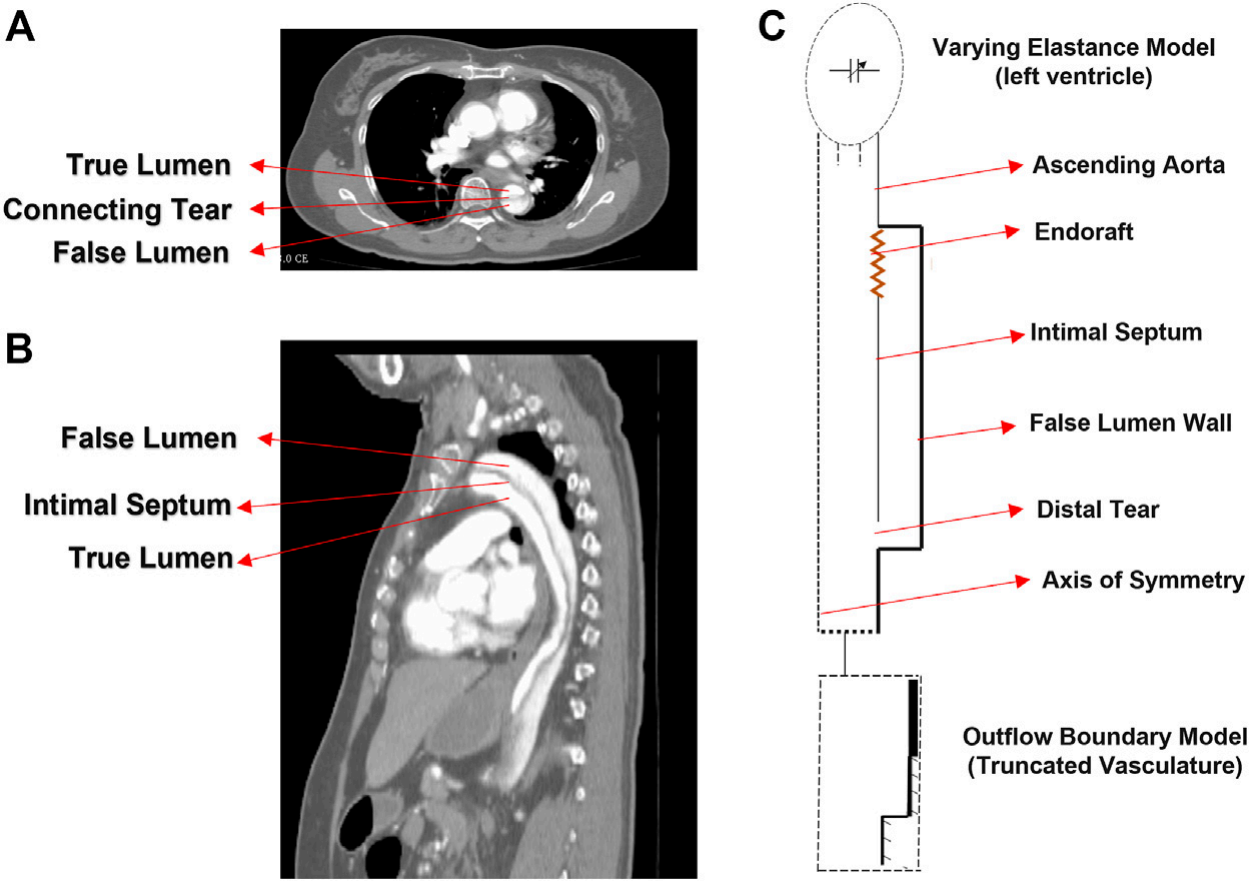
CT image **(A)** axial and **(B)** sagittal planes of the type B dissection patient. **(C)** Idealized model of type B aortic dissection with arrows indicating different segments of the model.

A schematic representation of the 3D axisymmetric model of the dissected aorta along with the illustrative images from TBAD patient is shown in Figure 1. In our idealized model, it is assumed that the TL is located concentric within the aorta and the FL is formed uniformly around the TL, connected with the flexible and compliant septum in the middle (Rudenick et al., 2015; Rudenick et al., 2017). For modeling the dynamics of the LV, the time-varying elastance model is used as an inlet condition of the dissection model (Amlani and Pahlevan, 2020). The importance of outflow boundary conditions to capture physiologically accurate hemodynamic waveforms is highlighted in the previous works (Olufsen, 1999; Karniadakis et al., 2005; Grinberg and Karniadakis, 2008). In this study, the extension tube boundary model (Pahlevan et al., 2011) was used as the outflow boundary condition to capture the compliance, resistance, and wave reflections of the downstream vasculature (Karreman, 1952; Aghilinejad et al., 2021b). The dimensions of the model are chosen within the average physiological range; the length of the TL is chosen from descending aorta to the bifurcation; and the length of the septum is chosen from descending aorta to renal arteries (Girish et al., 2016). The length of the endograft is varied in the range of 4–20 cm to cover the whole range of currently utilized endografts (Thrumurthy et al., 2011). To investigate the effect of endograft length in this study, endograft-septum length ratio ( λ ) is defined as $\text{λ}=\;\frac{\text{Graft}\;\text{Length}}{\text{Septum}\;\text{Length}}$ . Based on the utilized parameters in this study, λ∈(0.13,   0.26,  0.40,  0.53,  0.66) , where λ= 0.13 is considered to be short endograft, λ= 0.40 is considered to be medium endograft, and λ= 0.66 is considered to be long endograft. To account for the compliance mismatch between the replaced endograft and native aorta, the aortic wall and septum are considered to be compliant with stretching coefficient of the human aorta while the endograft is assumed to have a rigid wall. The physical parameters of this study are summarized in Table 1.

**TABLE 1:** Geometric and material parameters used in the computational models.

Name	Variable	Value	References
Length of the aortic model (cm)	L	40	Pahlevan and Gharib, (2011b)
Length of the septum (cm)	${\text{L}}_{\text{septum}}$	29	Williams et al. (1997)
Radius of the aorta (cm)	${\text{r}}_{\text{aorta}}$	1	Rudenick et al. (2013)
Bending coefficient of wall $\left(\text{Pa}\cdot {\text{m}}^{3}\right)$	EI	$2\times 10^{-7}$	Engelmayr et al. (2003)
Length of the outflow boundary model (cm)	${\text{L}}_{\text{boundary}}$	15	Pahlevan et al. (2011)
Contraction ratio of the rigid boundary model	κ	0.4	Pahlevan and Gharib, (2011a)
Volume compliance of the boundary model $\left({\text{m}}^{3}/\text{Pa}\right)$	${\text{C}}_{\text{outflow}}$	$3.14\times 10^{-11}$	Pahlevan et al. (2011)
LV compliance (ml/mmHg)	${\text{C}}_{\text{v}}\left(\text{t}\right)$	Figure 5	Berger et al. (1994)
LV dead volume (ml)	${\text{V}}_{\text{dead}}$	4	Amlani and Pahlevan, (2020)

### Mathematical Formulation

The immersed boundary-lattice Boltzmann method (IB-LBM) was used for the analysis of fluid flow with moving boundaries. To solve the pressure and flow fields in the fluid domain, a single-relaxation-time (SRT) incompressible LBM was used as an efficient solver of Navier–Stokes equations (Lee et al., 2005; Wang et al., 2015). In such a method, the synchronous motions of the particles on a regular lattice are enforced through a particle distribution function. This distribution function enforces mass and momentum conservation. It also ensures that the fluid is Galilean invariant and isotropic. The evolution of the distribution functions on the lattice is governed by the discrete Boltzmann equation with the BGK (Bhatnagar–Gross–Krook) collision model and the forcing term to couple the fluid and solid domains as

$${\text{f}}_{\text{i}}\left(x+e_{i}\Delta \text{t,}\;\text{t}+\Delta \text{t}\right)-{\text{f}}_{\text{i}}\left(x,\text{t}\right)=-\frac{1}{\text{τ}}\left[{\text{f}}_{\text{i}}\left(x,\text{t}\right)-{\text{f}}_{\text{i}}^{\text{eq}}\left(x,\text{t}\right)\right]+\Delta {\text{tF}}_{\text{i}},$$ (1)

where ${\text{f}}_{\text{i}}\left(x,\text{ t}\right)$ is the distribution function for particles with velocity $e_{\text{i}}$ at position x and time t. Δt and Δx are the time step and lattice space, respectively. The sound speed is $\text{c}=\;\frac{\Delta \text{x }}{\Delta \text{t}}=1$ . τ is a dimensionless relaxation time constant which is associated with fluid viscosity in the form $\text{μ}=\text{ρ}\vartheta ={\text{ρc}}_{\text{s}}^{2}\left(\text{τ}-\frac{1}{2}\right)\Delta \text{t}$ , where ϑ is the kinematic viscosity and ${\text{c}}_{\text{s}}=\frac{1}{\sqrt{3}}\text{c}$ is the lattice sound speed. The equilibrium distribution function for incompressible LBM and the forcing term are defined as

$${\text{f}}_{\text{i}}^{\text{eq}}={\text{ω}}_{\text{i}}{\text{ρ}}_{0}+{\text{ω}}_{\text{i}}\text{ρ}\left[\frac{e_{\text{i}}\cdot v}{{\text{ c}}_{\text{s}}^{2}}+\frac{{\left(e_{\text{i}}\cdot v\right)}^{2}}{\;2{\text{c}}_{\text{s}}^{4}}-\frac{v^{2}}{\;2{\text{c}}_{\text{s}}^{2}}\right],$$ (2)

$${\text{F}}_{\text{i}}=\left(1-\frac{1}{2\text{τ}}\right){\text{ω}}_{\text{i}}\left(\frac{e_{\text{i}}-v}{{\text{ c}}_{\text{s}}^{2}}+\frac{e_{\text{i}}\cdot v}{{\text{c}}_{\text{s}}^{4}}e_{\text{i}}\right)\cdot f\text{,}$$ (3)

where ${\text{ω}}_{\text{i}}$ is the weighting factor, ${\text{ρ}}_{0}$ is related to the pressure by ${\text{ρ}}_{0}=\frac{\text{p}}{{\text{c}}_{\text{s}}^{2}}$ , f is the force density at the Eulerian point, and velocity v can be calculated by

$${\text{ρ}}_{0}=\sum {\text{f}}_{\text{i}},$$ (4)

$$\text{ρ}v=\sum e_{\text{i}}{\text{f}}_{\text{i}}+\frac{1}{2}\;f\Delta \text{t}\text{.}$$ (5)

At the interface of the aortic and septal wall with the fluid, the IB algorithm was used, and the bounce-back boundary condition was used for modeling the fluid flow at the interface of the rigid boundary (endograft). A source term was considered (Lee et al., 2005) to satisfy the axisymmetric condition at the centerline (Bilgi and Atalık, 2020). To compute the deformation of the elastic aortic and septum wall, the dynamic motion of these two in the Lagrangian form is solved using

$${\text{ρ}}_{\text{s}}\text{h}\frac{\partial^{2}X}{\partial {\text{t}}^{2}}=\frac{\partial }{\partial \text{s}}\left[\text{Eh}\left(1-{\left(\frac{\partial X}{\partial \text{s}}\cdot \frac{\partial X}{\partial \text{s}}\right)}^{-1/2}\right)\frac{\partial X}{\partial \text{s}}-\;\frac{\partial }{\partial \text{s}}\left(\text{EI}\frac{\partial^{2}X}{\partial {\text{s}}^{2}}\right)\right]+F_{\text{L}}\text{,}$$ (6)

where s is the arclength of the wall, h is the thickness, X=(X(s,t),Y(s,t)) is the position of the wall, ${\text{ρ}}_{\text{s}}$ is the density of the aortic and septum wall, Eh is the stretching stiffness, EI is the bending stiffness, and $F_{\text{L}}$ is the Lagrangian force exerted on the wall by the surrounding fluid. The simple support boundary condition applied at the fixed points of the two sides of the septum wall (Huang et al., 2007), which is given by

$$X=X_{0},\frac{\partial^{2}X}{\partial {\text{s}}^{2}}=\left(0,0\right).$$ (7)

**FIGURE 2:**
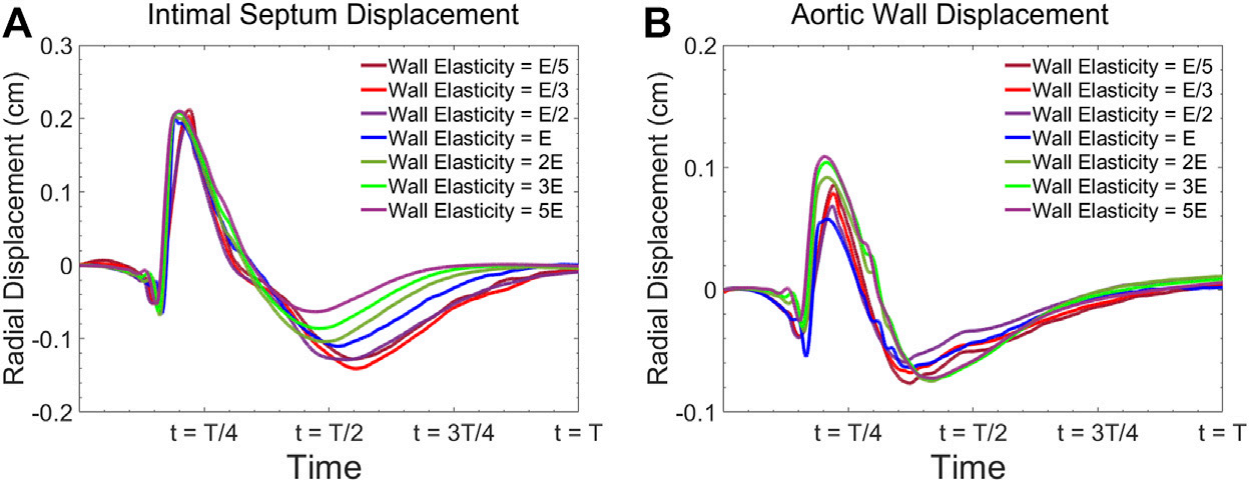
Radial vessel wall displacement waveform at the center of the model for **(A)** intimal septum, and **(B)** the aortic wall for various elasticities.

For the same geometrical configuration, the material parameter which affects the deformation of the vessel wall governed by Eq 6 is only the material elasticity ( E ). Since there is a range for reported physiological values for vessel wall elasticity and also there are uncertainties in determining the septum properties, it is essential to investigate the impact of selected material parameter on the solution of the dynamical model (Eq 6). Figure 2 shows the sensitivity analysis of the radial displacement of both the intimal septum and aortic wall computed at the center of the model during one cardiac cycle with different material elasticities. While the results show our model is able to capture the effect of elasticity on dynamic motion of the wall, the overall shape of the displacement waveform for different elasticities is preserved. In this study, we used the baseline parameters reported in Table 1.

### Implementations of the Boundary Conditions

The LV was modeled as a time-varying compliance model coupled with the aorta. The extension tube outflow boundary model was used for the truncated vasculature at the outlet of our 3-D FSI solver. At the inlet, the pressure ${\text{p}}_{\text{v}}\left(\text{t}\right)$ inside the LV and the corresponding volume ${\text{V}}_{\text{v}}\left(\text{t}\right)$ in the LV are connected via time-varying compliance ${\text{C}}_{\text{v}}\left(\text{t}\right)$ given by

$${\text{V}}_{\text{v}}\left(\text{t}\right)-{\text{V}}_{\text{dead}}={\text{C}}_{\text{v}}\left(\text{t}\right){\text{p}}_{\text{v}}\left(\text{t}\right).$$ (8)

In Eq 8, the constant ${\text{V}}_{\text{dead}}$ known as the dead volume is the limit for pressure generation. Substituting the relation between the flow into the aorta with the ${\text{V}}_{\text{v}}\left(\text{t}\right)$ and differentiating Eq 8 with respect to t , we can get the following ordinary differential equation (ODE) for the pressure inside the LV

$$\frac{\partial {\text{p}}_{\text{v}}\left(\text{t}\right)}{\partial \text{t}}=-\frac{1}{{\text{C}}_{\text{v}}\left(\text{t}\right)}\left[\frac{\partial {\text{C}}_{\text{v}}\left(\text{t}\right)}{\partial \text{t}}{\text{p}}_{\text{v}}\left(\text{t}\right)+\text{Q}\left(\text{x}=0,\text{t}\right)\right].$$ (9)

**FIGURE 3:**
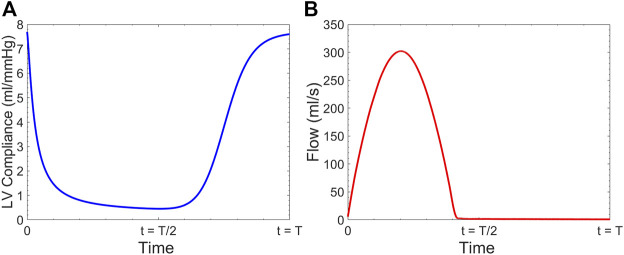
**(A)** Time-varying left ventricular compliance $C_{v}\left(t\right)$ , and **(B)** computed flow waveform at the aortic root using our computational model.

Clinically, ${\text{C}}_{\text{v}}\left(\text{t}\right)$ stands for inverse of LV end-systolic elastance ( ${\text{E}}_{\text{es}}$ ) which is the measure of LV contractility (Amlani and Pahlevan, 2020; Berger et al., 1994) (Figure 3A). Once ${\text{P}}_{\text{v}}\left(\text{t}\right)$ is greater than the pressure at the interface of the aorta and the LV, the valve opens and $\text{p}\left(\text{x}=0,\text{t}\right)={\text{p}}_{\text{v}}\left(\text{t}\right)$ with the flow condition given by the fluid solver (the ODE condition). Once the inflow reaches zero (or, numerically, the time at which Q(x=0,t)≤0 ), the valve closes, and the left boundary condition remains Q(x=0,t)=0 (a Dirichlet-type condition). Figure 3A shows the empirically given time-varying compliance ( ${\text{C}}_{\text{v}}\left(\text{t}\right)$ ) reported from clinical data for normal contractile state of LV (Berger et al., 1994). Figure 3B demonstrates the computed sample flow response to the LV model at the aortic root in our model resulting in 5.7 L/min for average cardiac output (CO) over the cycle T .

At the terminal boundary x=L , the physical outflow boundary model approximates the effect of the truncated vasculature and peripheral vessels. This extension tube boundary model is a simple outflow boundary condition for three-dimensional fluid-structure interaction (FSI) simulation of pulsatile blood flow in compliant vessels. In this structural model, the computational domain is extended with an elastic tube connected to a rigid contraction to account for the compliance, resistance, and the wave reflection of the truncated vascular network. Previous studies showed that this model is suitable for cardiac transient (non-periodic) events (Pahlevan et al., 2011; Kang et al., 2019). The parameters of the outflow boundary condition model are given in Table 1, where the contraction ratio κ is the ratio of the radius of the rigid boundary tube (after the contraction) to the original radius (before the contraction). The presence of the rigid contraction is more attributed to the required resistance for the system, while the elastic portion accounts for the compliance of the eliminated vasculature. These parameters are chosen based on the data in the literature to physiologically capture wave dynamics in the aorta (Pahlevan et al., 2011; Kang et al., 2019).

### Numerical Method

The D2Q9 velocity model is applied in the LBM with the sound speed c where the velocity set is given by

$$e_{\text{i}}=\left\{ \begin{array}{l} 0\;\;i=0\\ \left(\cos\left[\left(\text{i}-1\right)\frac{\text{π}}{2}\right],\;\sin\left[\left(\text{i}-1\right)\frac{\text{π}}{2}\right]\right)c\;\;i=1,2,3,4\\ \sqrt{2}\left(\cos\left[\left(\text{i}-5\right)\frac{\text{π}}{2}+\frac{\text{π}}{4}\right],\sin\left[\left(\text{i}-5\right)\frac{\text{π}}{2}\right]+\frac{\text{π}}{4}\right)c\;\;i=5,6,7,8 \end{array}\right..$$ (10)

Axisymmetric LBM is implemented in this study using an incompressible D2Q9 BGK model. In pseudo-Cartesian coordinates (x,r) for describing 3D axisymmetric flow, Eq 1 can be transformed into

$${\text{f}}_{\text{i}}\left(\text{x}+{\text{e}}_{\text{i}}\text{Δt},\text{t}+\text{Δt}\right)-{\text{f}}_{\text{i}}\left(\text{x},\text{t}\right)=-\frac{1}{\text{τ}}\left[{\text{f}}_{\text{i}}\left(\text{x},\text{t}\right)-{\text{f}}_{\text{i}}^{\text{eq}}\left(\text{x},\text{t}\right)\right]+{\text{ΔtF}}_{\text{i}}\left(\text{x},\text{t}\right)+{\text{H}}_{\text{i}}\left(\text{x},\text{t}\right),$$ (11)

where a source term ${\text{H}}_{\text{i}}\left(\text{x},\text{t}\right)$ is given by

$${\text{H}}_{\text{i}}\left(\text{x},\text{t}\right)={\text{Δth}}_{\text{i}}^{\left(1\right)}\left(\text{x},\text{t}\right)+{\text{Δt}}^{2}{\text{h}}_{\text{i}}^{\left(2\right)}\left(\text{x},\text{t}\right),$$ (12)

$${\text{h}}_{\text{i}}^{\left(1\right)}=-\frac{{\text{ω}}_{\text{i}}{\text{ρv}}_{\text{r}}}{\text{r}},$$ (13)

$${\text{h}}_{\text{i}}^{\left(2\right)}=-{\text{ω}}_{\text{i}}\frac{3\text{ν}}{\text{r}}\left[\partial_{\text{y}}\text{P}+\text{ρ}\partial_{\text{x}}{\text{v}}_{\text{x}}{\text{v}}_{\text{r}}+\text{ρ}\partial_{\text{r}}{\text{v}}_{\text{r}}{\text{v}}_{\text{r}}+\text{ρ}\left(\partial_{\text{r}}{\text{v}}_{\text{x}}-\partial_{\text{x}}{\text{v}}_{\text{r}}\right){\text{e}}_{\text{ix}}\right].$$ (14)

${\text{H}}_{\text{i}}\left(\text{x},\text{t}\right)$ is the added source term into the collision step defined based on ${\text{h}}_{\text{i}}^{\left(1\right)}$ and ${\text{h}}_{\text{i}}^{\left(2\right)}$ with $\text{P}={\text{c}}_{\text{s}}^{2}\cdot {\text{ρ}}_{\text{o}}$ . The source term is added to recover the extra terms caused by the curvature from the continuity equation and Navier–Stokes equation in cylindrical coordinates (Lee et al., 2005; Huang and Lu, 2009). For calculating the derivatives of the velocity vector along the radial and axial directions, the terms $\partial_{\text{r}}{\text{v}}_{\text{x}}+\partial_{\text{x}}{\text{v}}_{\text{r}}$ , $\partial_{\text{x}}{\text{v}}_{\text{x}}$ , and $\partial_{\text{r}}{\text{v}}_{\text{r}}$ can be obtained by the following equation (Lee et al., 2005):

$$\text{ρν}\left(\partial_{\text{β}}{\text{v}}_{\text{α}}+\partial_{\text{α}}{\text{v}}_{\text{β}}\right)=-\left(1-\frac{1}{2\text{τ}}\right)\sum_{\text{i}=0}^{8}\left({\text{f}}_{\text{i}}-{\text{f}}_{\text{i}}^{\text{eq}}\right){\text{e}}_{\text{iα}}{\text{e}}_{\text{iβ}}+\text{o}\left({\text{ε}}^{2}\right),$$ (15)

where substituting α=x and β=r gives us a relation for $\partial_{\text{r}}{\text{v}}_{\text{x}}+\partial_{\text{x}}{\text{v}}_{\text{r}}$ ; substituting α=β=x gives us a relation for $\partial_{\text{x}}{\text{v}}_{\text{x}}$ ; and substituting α=β=r gives us a relation for $\partial_{\text{r}}{\text{v}}_{\text{r}}$ . For calculating $\partial_{\text{r}}{\text{v}}_{\text{x}}-\partial_{\text{x}}{\text{v}}_{\text{r}}$ in Eq 14 the only value left unknown is $\partial_{\text{x}}{\text{v}}_{\text{r}}$ . Below is a finite difference method employed to obtain $\partial_{\text{x}}{\text{v}}_{\text{r}}$ at lattice node (i,j) with the following expression:

$${\left(\partial_{\text{x}}{\text{v}}_{\text{r}}\right)}_{\left(\text{i},\text{j}\right)}=\frac{{\left({\text{v}}_{\text{r}}\right)}_{\left(\text{i}+1,\text{j}\right)}-{\left({\text{v}}_{\text{r}}\right)}_{\left(\text{i}-1,\text{j}\right)}}{2\Delta \text{x}}.$$ (16)

The solid deformation equation (Eq 6) was solved by the finite element method (FEM) (Doyle, 2001). The IB method was used to couple the fluid and solid solvers. Particularly, implicit velocity correction-based IB approach was used in this study which has been extensively used to simulate the FSI problems in cardiovascular biomechanics (Peskin, 2002; Mittal and Iaccarino, 2005). In this method, the body force term f is used as an interaction force between the fluid and the boundary to enforce the no-slip velocity boundary condition by introducing an intermediate velocity $v^{*}$ by

$$v\left(x,\text{t}\right)=v^{\text{∗}}\left(x,\text{t}\right)+\text{δ}v\left(x,\text{t}\right).$$ (17)

The relation between the velocity correction δv and the body force term f is

$$\text{ρδ}v\left(x,t\right)=\frac{1}{2}f\left(x,t\right)\text{δ}t\text{.}$$ (18)

While in the conventional IBM, f is computed in advance and then the velocity correction δv and corrected velocity v(x,t) are explicitly computed, there is no guarantee the velocity at the boundary satisfies the no-slip boundary condition (Wu and Shu, 2009). In the revised implicit velocity correction-based immersed boundary approach, the velocity correction δv term at the Eulerian point (fluid domain) can be first obtained by the following Dirac delta function interpolation as

$$\text{δ}v\left(x,\text{t}\right)=\underset{\text{Γ}}{\int }\text{δ}V\left(\text{s},\text{t}\right)\text{δ}\left(x-X\left(\text{s},\text{t}\right)\right)\text{ds,}$$ (19)

where δ(x−X(s,t)) is smoothly approximated by a continuous kernel distribution and δV(s,t) is the unknown velocity correction vector at every Lagrangian point at the FSI boundary Γ as proposed by previous works (Wu and Shu, 2009). Note that in the notation above, x is the Eulerian coordinates related to the fluid phase while X stand for Lagrangian coordinates related to the solid phase. In order to meet the non-slip boundary condition, the fluid velocity at the boundary point Ω obtained by the smooth δ function interpolation must be equal to the wall velocity V at the same position. Its mathematical expression is

$$V\left(\text{s},\text{t}\right)=\;\underset{\text{Ω}}{\int }v\left(x,\text{t}\right)\text{δ}\left(x-X\left(\text{s},\text{t}\right)\right)\text{d}x\text{.}$$ (20)

Substituting Eqs. 17–20, we can get the following equation:

$$V\left(\text{s},\text{t}\right)=\;\underset{\text{Ω}}{\int }v^{\text{∗}}\left(x,\text{t}\right)\text{δ}\left(x-X\left(\text{s},\text{t}\right)\right)\text{d}x+\underset{\text{Ω}}{\int }\left[\underset{\text{Γ}}{\int }\text{δ}V\left(\text{s},\text{t}\right)\text{δ}\left(x-X\left(\text{s},\text{t}\right)\right)\text{ds}\right]\text{δ}\left(x-X\left(\text{s},\text{t}\right)\right)\text{d}x\text{.}$$ (21)

where the only unknown velocity correction δV(s,t) can be obtained by solving this equation. In the utilized IB approach, after determining the velocity correction terms via Eq 17, the force density acting on the fluid phase f can be calculated using Eq 18. Lastly, the boundary force density at Lagrangian points $F_{\text{L}}$ can be explicitly found by

$$F_{\text{L}}\left(\text{s},\text{t}\right)=-\underset{\text{Ω}}{\int }f\left(x,\text{t}\right)\text{δ}\left(x-X\left(\text{s},\text{t}\right)\right)\text{d}x\text{.}$$ (22)

**FIGURE 4:**
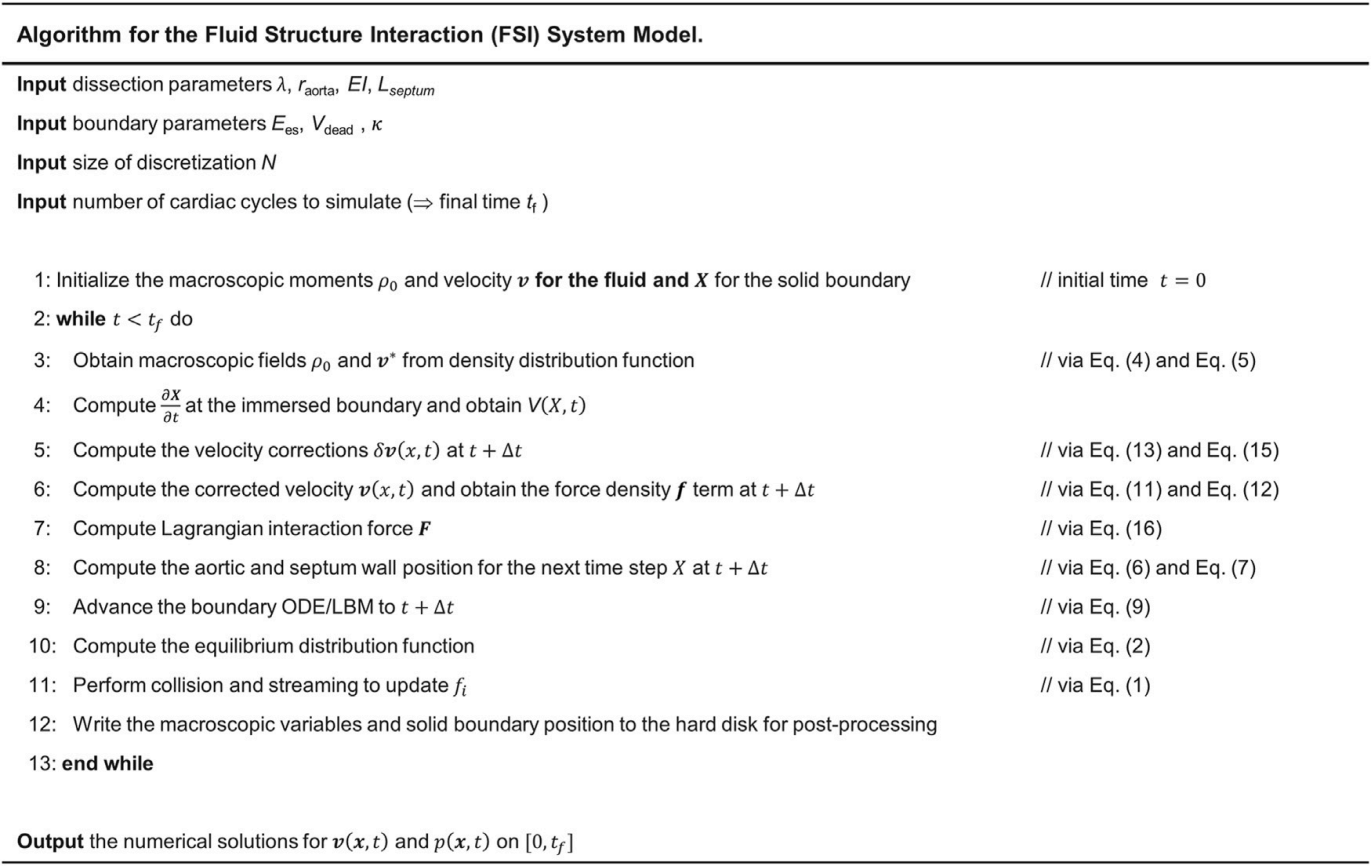
Steps for implementation of IB-LBM-FEM algorithm to numerically solve the LV-Dissection system model with time-varying elastance LV input.

The clinical and physical quantities were connected to the numerical quantities using dimensionless parameters including the Womersley number $\text{Wo}={\text{r}}_{\text{aorta}}\sqrt{\frac{\text{αρ}}{\text{μ}}}$ where ${\text{r}}_{\text{aorta}}$ is the reference length (radius of the aorta) and α is the pulsation frequency (i.e., heart rate) (Doyle, 2001; Huang et al., 2018). For spatial and temporal discretization, each simulation was run at $\frac{\text{D}}{\Delta \text{x}}=32$ with a time step of $\frac{\text{T}}{\text{Δt}}=50,000\left(\text{T}=2\cdot \text{π}/\text{α}\right)$ . Mesh independence studies are done on the pressure profiles at different cross-sections of the model to ensure that this mesh density and time step are sufficient for the accurate calculations. Simulations were run on USC’s center for advanced research computing cluster nodes, each node equipped with 20 cores (2,600 MHz) with 64 GB memory. At least 10 cardiac cycles were simulated to ensure a periodic steady state was reached. The complete FSI solver for the LV-dissection model is summarized in the pseudo-code of the algorithm shown in Figure 4.

### Hemodynamic Analysis

The pulsatile power ( ${\bar{\text{P}}}_{\text{pulse}}$ ) was used in this study to quantify the LV power requirement. ${\bar{\text{P}}}_{\text{pulse}}$ is the difference between the total power ${\bar{\text{P}}}_{\text{total}}$ and the steady power ${\bar{\text{P}}}_{\text{s}}$ . The total power was calculated based on the average product of the pressure p(t) and flow q(t) during one cardiac cycle T , while steady power was calculated based on the product of the average pressure and average flow during a cardiac cycle. Each of these power quantities are respectively given by

$${\bar{\text{P}}}_{\text{total}}=\frac{1}{\text{T}}\int_{\text{0}}^{\text{T}}\text{p}\left(\text{t}\right)\text{q}\left(\text{t}\right)\text{dt},$$ (23)

$${\bar{\text{P}}}_{\text{s}}={\text{p}}_{\text{mean}}{\text{q}}_{\text{mean}},$$ (24)

$${\bar{\text{P}}}_{\text{pulse}}={\bar{\text{P}}}_{\text{total}}-{\bar{\text{P}}}_{\text{s}}.$$ (25)

Reverse Flow Index (RFI) is calculated to quantify the flow reversal as a measure to predict thrombose formation, following the works done by Birjiniuk et al. (Birjiniuk et al., 2017; Birjiniuk et al., 2019; Birjiniuk et al., 2020). RFI is defined as the ratio of the retrograde flow ${\text{Q}}_{\text{reverse}}$ (which is in the opposite direction of the systemic circulation) over the absolute summation of the antegrade flow ${\text{Q}}_{\text{forward}}$ (which is in the same direction of the systemic circulation) and retrograde flow, given by

$$\text{RFI}=\frac{\left|\int_{\text{0}}^{\text{T}}{\text{Q}}_{\text{reverse}}\text{dt}\right|}{|\int_{\text{0}}^{\text{T}}{\text{Q}}_{\text{reverse}}\text{dt}|+\;\left|\int_{\text{0}}^{\text{T}}{\text{Q}}_{\text{forward}}\text{dt}\right|}\times 100.$$ (26)

**FIGURE 5:**
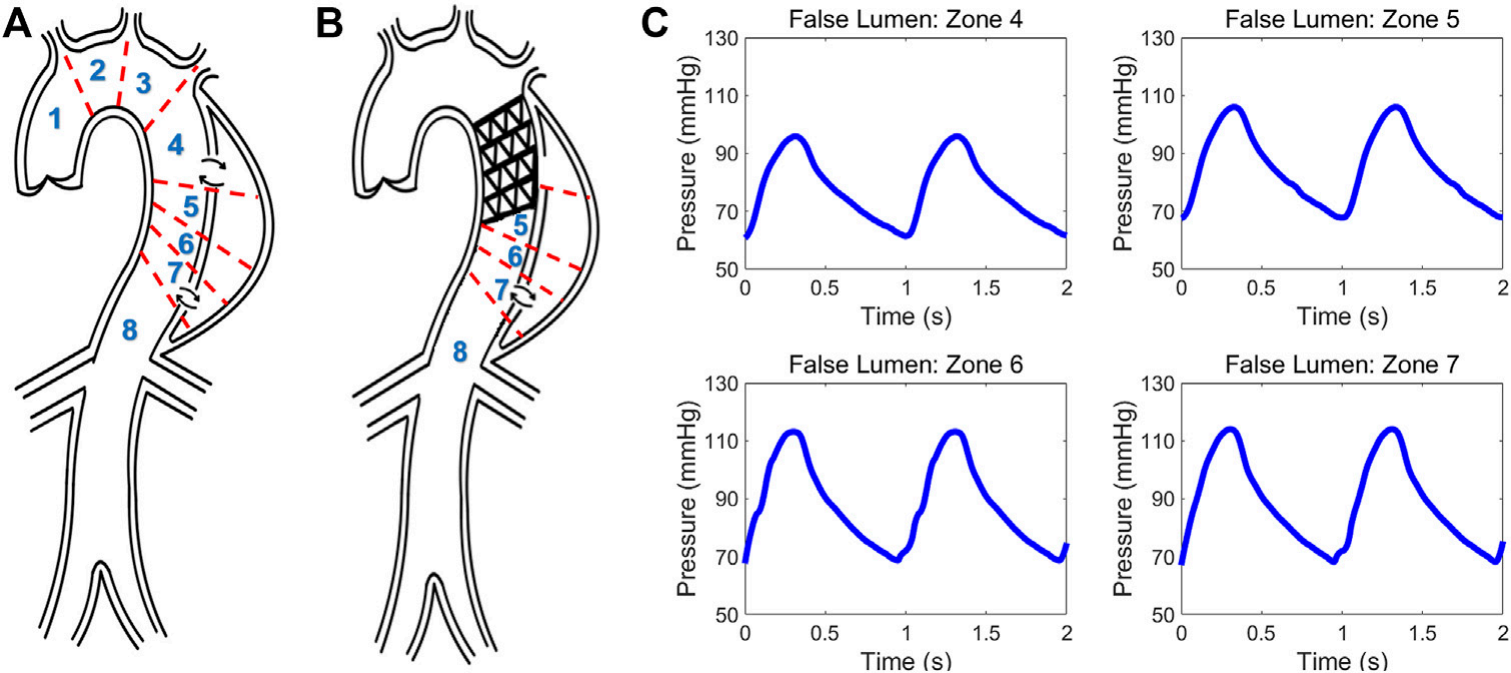
Schematic of type B aortic dissection **(A)** pre- and **(B)** post-TEVAR. Different zones are classified for computing hemodynamic quantities. **(C)** Invasive pressure data measured post-TEVAR via the ComboMap system from the patient.

To quantify ${\text{Q}}_{\text{reverse}}$ and ${\text{Q}}_{\text{forward}}$ , velocity profiles in each lumen were integrated across luminal cross-sections at different zones (Figures 5A, B) at each cardiac phase.

### Patient Description and Invasive Clinical Measurement

Data from a TBAD patient undergoing TEVAR was studied and utilized to examine the physiological accuracy of our model. The participant was provided with written informed consent and all protocols were approved by the Keck Medical Center of the University of Southern California (USC) Institutional Review Board. The dissection started distal to the origin of the left subclavian artery and extended to the infrarenal aorta and the TEVAR endograft extended from proximal to the left subclavian to the mid-descending thoracic aorta. The entire patient’s aorta was imaged before and after the TEVAR with computed tomography angiography (CTA) with 1 mm slices, and illustrative images in the axial and sagittal planes are shown in Figures 1A, B. The ComboMap system with a ComboWire guide wire (Philips Volcano Corporation) was used to acquire pressure and flow data inside the TL and FL. The guide wire was 0.36 mm in diameter and 185 cm in length. The sensor contained a pressure transducer and an ultrasound transducer, both mounted in a single housing at the tip of the guide wire. Data collected during invasive assessment were extracted directly from the ComboMap system at 200 Hz sampling rate. The measurements were done at all different aortic zones as demonstrated in Figures 5A, B. Samples of the invasive measured pressure waveforms are shown in Figures 5C at different zones inside the FL post-TEVAR.

## Results

### Physiological Accuracy of the Model

**FIGURE 6:**
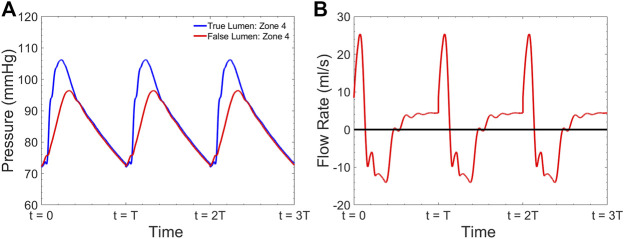
Simulated **(A)** pressure inside the TL and FL at zone 4 and **(B)** flow inside the FL at zone 4 for λ = 0.66 and HR = 60 bpm.

A sample pressure inside the TL and FL at zone 4 is shown in Figure 6A. The expected fiducial features of the pressure wave inside the TL including the pressure dicrotic notch can be seen in this Figure. The shape of the FL pressure waveform matches well with the measured data shown in Figure 5C. Figure 6B demonstrates the computed flow waveform inside the FL at the place where the endograft is implanted. The flow pattern consists of systolic biphasic flow which is similar to the findings of Rudenik et al. (Rudenick et al., 2017) who reported the phase-contrast magnetic resonance imaging of 31 patients with AD.

Table 2 presents the comparison between the results of our computational model with our measured invasive clinical data. Note that the Womersley number and the endograft-septum length ratio are matched in accordance with the clinical values based on patient’s characteristics ( Wo ≈ 11.2) and TEVAR procedure ( λ ≈ 0.66). Relative pulse pressure (RPP) inside the FL is used to compare the computational and clinical data, defined as $\text{RPP}\left(\text{i},\text{j}\right)\;=\;\frac{{\text{pp}}_{{\text{zone}}_{\text{i}}}-{\text{pp}}_{{\text{zone}}_{\text{j}}}}{{\text{pp}}_{{\text{zone}}_{\text{j}}}}$ for i=5, 6, 7 , and j=4 . This hemodynamic parameter is related to the overall fluid motion inside the FL, and it is controlled more by the underlying physics rather than the patient-specific geometry. Therefore, it is suitable to be utilized for the comparison in this study.

**TABLE 2:** Comparison between invasive clinical measurements and the results from our computational model.

Hemodynamic Variable	RPP(4,5)	RPP(4,6)	RPP(4,7)
Measurement Type			
Invasive Clinical Data	0.059	0.088	0.294
FSI Computational Model	0.044	0.073	0.327

**FIGURE 7:**
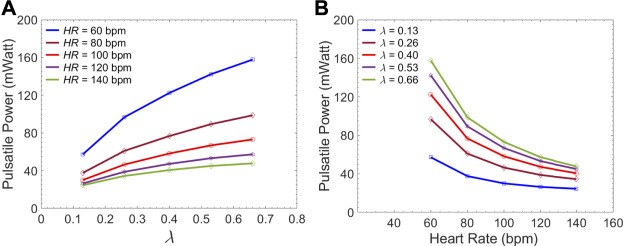
Average LV pulsatile power requirement per cardiac cycle versus **(A)** the λ (endograft-septum length ratio) at different HRs and versus **(B)** the HR at different λ .

* $\text{RPP}\left(\text{i},\text{j}\right)\;=\;\frac{{\text{pp}}_{{\text{zone}}_{\text{i}}}-{\text{pp}}_{{\text{zone}}_{\text{j}}}}{{\text{pp}}_{{\text{zone}}_{\text{j}}}}$ are calculated for comparing the clinical and computational data. Zones’ classification is illustrated in Figure 7A.

### Effect of Endograft Length on Left Ventricular Workload

Figure 7A gives the left ventricular pulsatile power requirement ${\bar{\text{P}}}_{\text{pulse}}$ as a function of the endograft-septum length ratio ( λ ) for different heart rates (HRs). In these cases, the CO of the LV is kept constant at the value of 5.7 L/min . The calculated pulsatile power is based on the pressure and flow data at Zone 1 in the TL. As expected, LV pulsatile power increases at all HRs when the endograft length increases. Figure 7B demonstrates the left ventricular pulsatile power requirement as a function of HRs for different endograft-septum length ratios.

### Effect of Endograft Length on FL Flow Reversal

**FIGURE 8:**
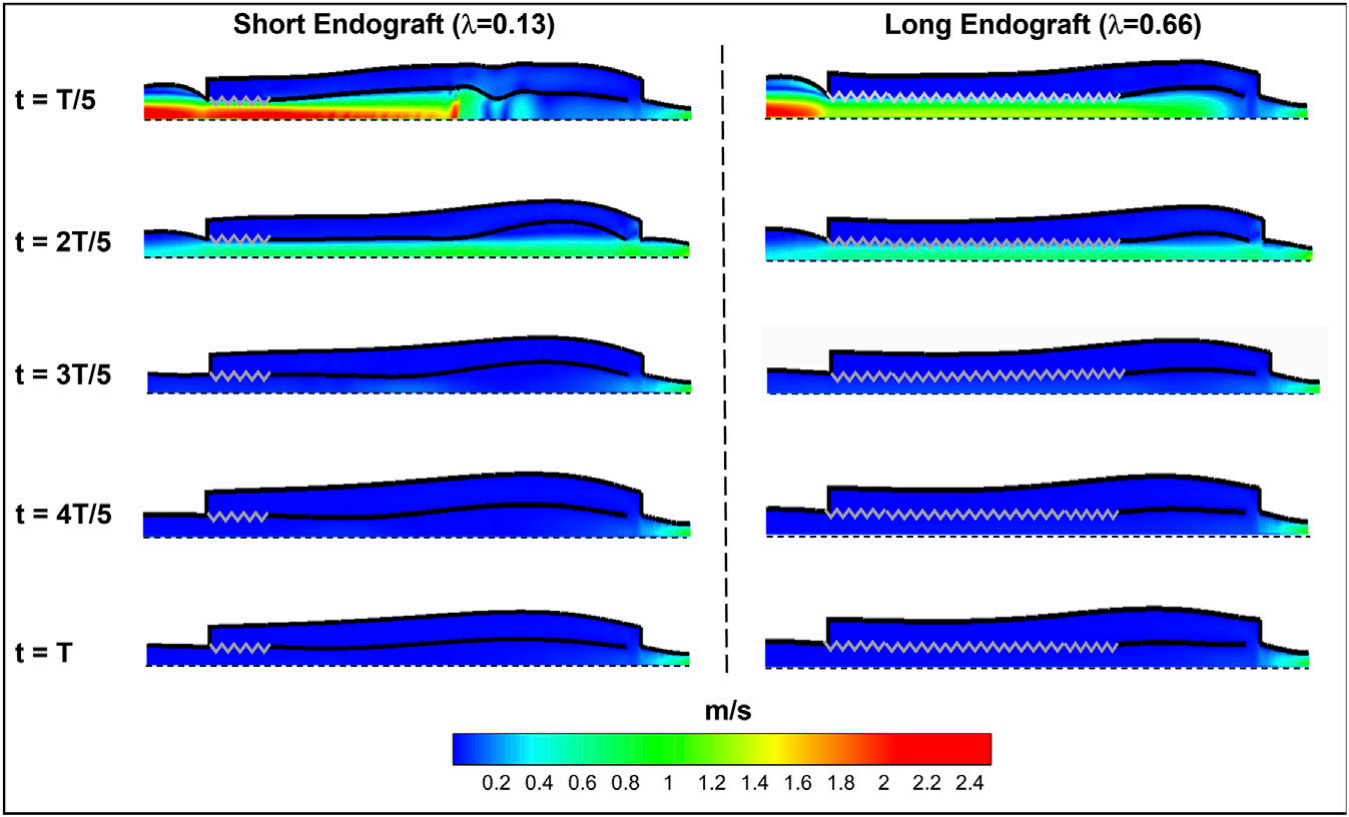
Spatial distribution of fluid and solid behavior in the FSI type-B dissection model at various times during the cardiac cycle. The zig-zag boundary shows the graft (rigid) schematically and the dashed wall represent the axis of the symmetry. The flow direction is from left to the right.

**FIGURE 9:**
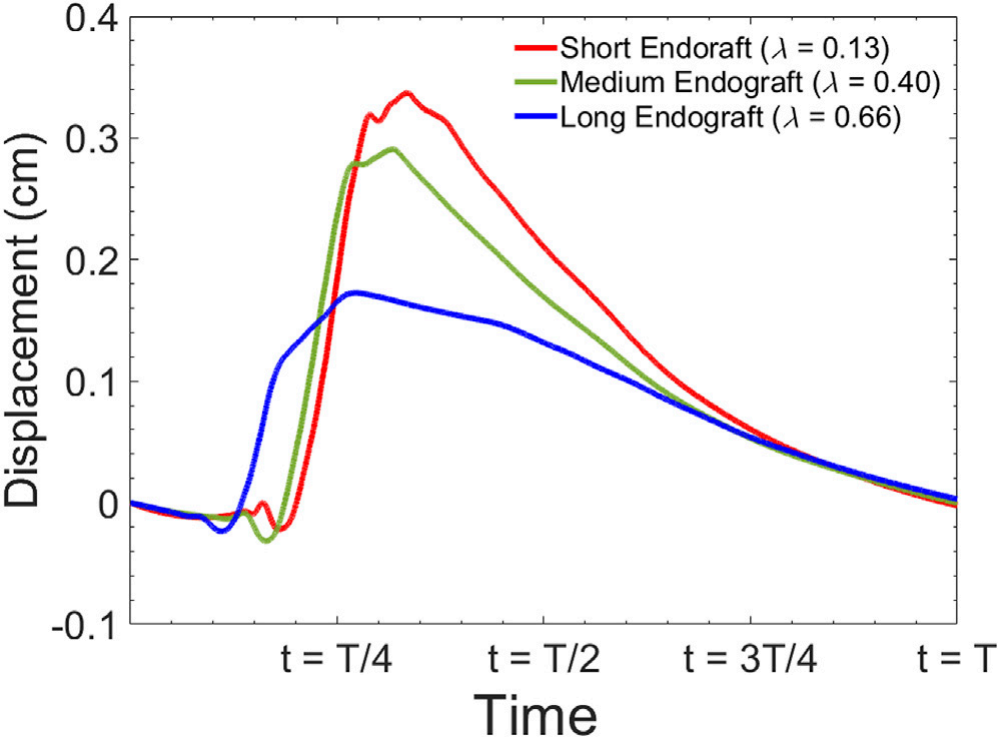
Simulated septum wall displacement waveform for different graft lengths during one cardiac cycle. The data are collected 5 cm proximal to the distal tear.

Figure 8 presents the fluid velocity amplitudes in the fluid domain as well as the septum and aortic wall displacements at various snapshots in time during a cardiac cycle of length T for short and long grafts. The displacement waveform of the intimal septum 5 cm proximal to the distal tear in the presence of short, medium, and long endografts is shown in Figure 9.

**FIGURE 10:**
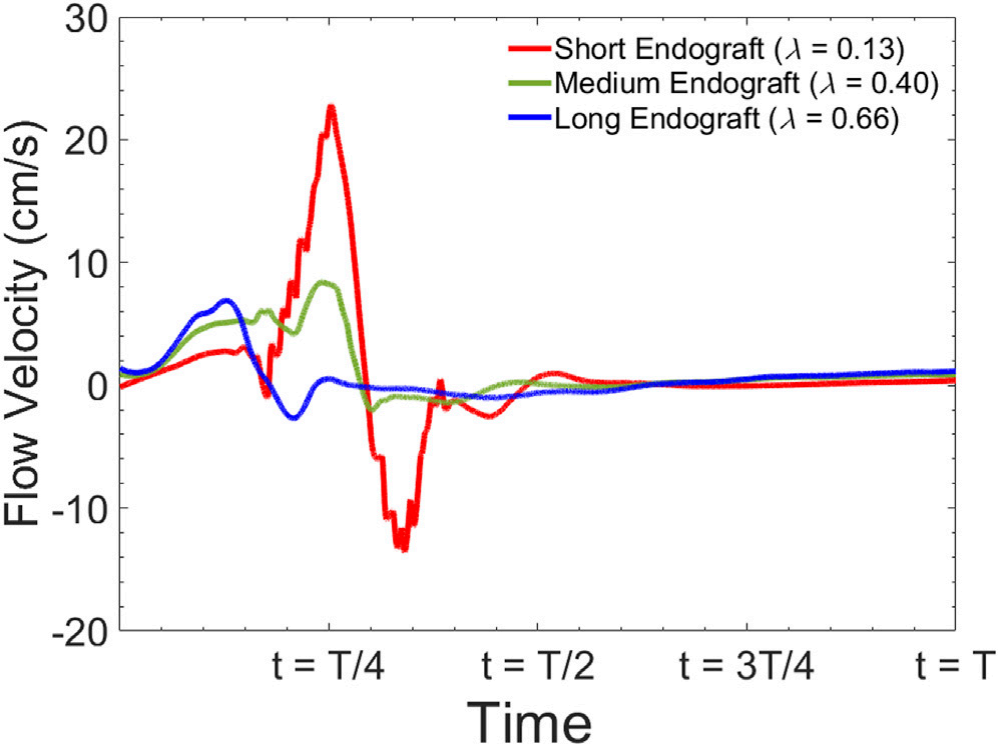
Simulated flow velocity waveform inside the false lumen for different graft lengths during one cardiac cycle. The data are collected 5 cm proximal to the distal tear.

**FIGURE 11:**
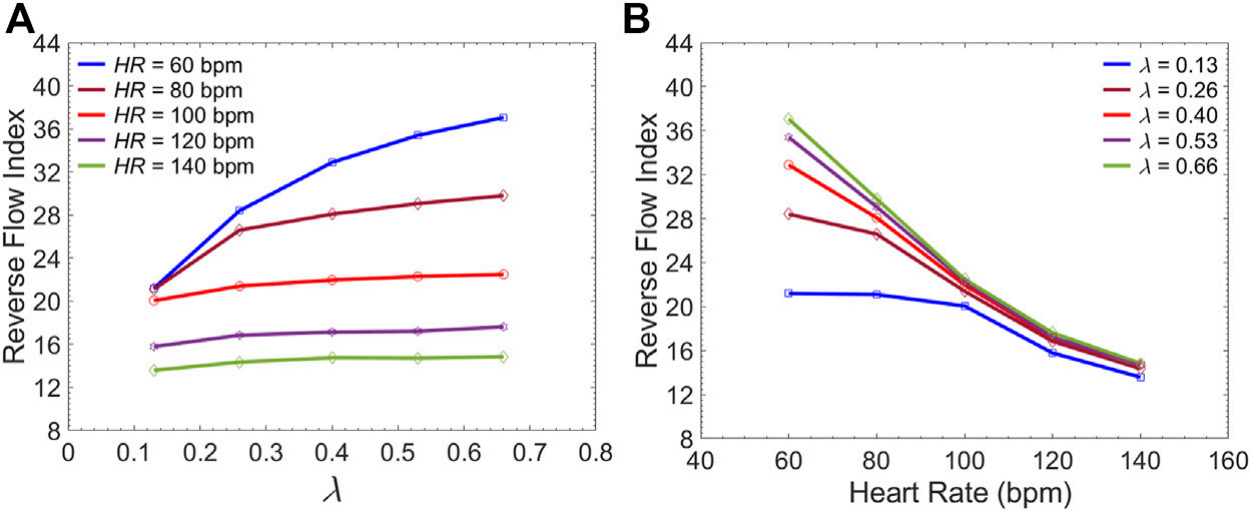
Average Reverse Flow Index inside the FL per cardiac cycle versus **(A)** the λ (endograft-septum length ratio) at different HRs and versus **(B)** the HR at different λ .

Figure 10 presents the sample of velocity profile inside the FL for short, medium and long endografts. The velocity is computed at the center of the of the FL 5 cm proximal to the distal tear. Figure 11A demonstrates RFI (to quantify FL flow reversal) as a function of λ for different HRs. RFI is reported based on the average of the values computed at Zones 4, 5, and 6 inside the FL (Figure 3B). Similar to the previous section, the CO of the LV is kept constant at the value of 5.7 L/min . Figure 11B shows RFI as a function of HRs for different endograft-septum length ratios.

### Effect of LV Contractile State on FL Flow Reversal

**FIGURE 12:**
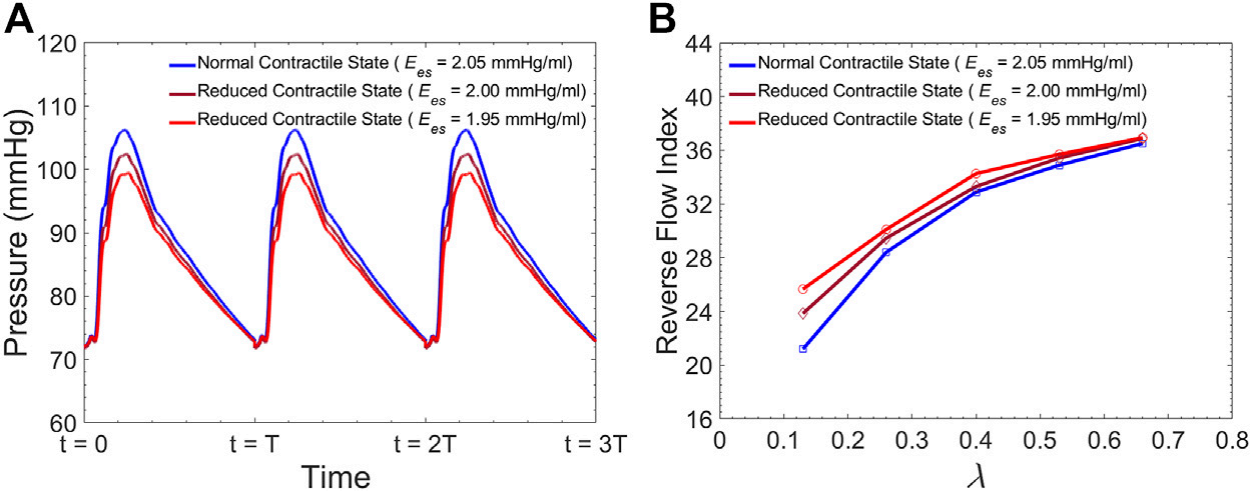
**(A)** Simulated pressure inside the TL at zone 4 for different levels of LV contractility, and **(B)** Average Reverse Flow Index inside the FL per cardiac cycle versus the λ (endograft-septum length ratio) at different levels of LV contractility. $E_{es}=2\text{.}05\text{ mmHg}/\text{ml}$ corresponds to CO=5.7l/min , $E_{es}=2.00\text{ mmHg/ml}$ corresponds to CO=5.2l/min , and $E_{es}=1\text{.}95\text{ mmHg}/\text{ml}$ corresponds to CO=4.7l/min .

Figure 12A demonstrates the pressure inside the TL at zone 4 for three different LV contractility demonstrated by ${\text{E}}_{\text{es}}$ . Figure 12B presents RFI as a function of endograft-septum length ratio ( λ ) for these three different contractile states of the left ventricle ( ${\text{E}}_{\text{es}}=2.05\text{mmHg}/\text{ml}$ corresponds to CO=5.7L/min ). These simulations run at fixed HR of 60 bpm .

## Discussion

In this study, we investigated clinically relevant hemodynamic patterns inside the TL and FL after endovascular repair using a physiologically accurate idealized model of TBAD. Our results suggest that: 1) There is a non-linear trend towards increased FL flow reversal as the endograft length increases but with an increased LV workload, 2) at a given heart cardiac output, lower HR enhances FL flow reversal and recirculation independent of the endograft length, and 3) at a given HR, a reduced LV contractility enhances FL flow reversal and reduces the systolic blood pressure.

### Model Validation Against Invasive Clinical Measurements

We utilized FSI computational model of the coupled LV-aorta system to gain insight on the biomechanical behavior of blood flow in type B dissection following TEVAR. Numerous computational models, both patient-specific and lumped parameter (Cheng et al., 2015; Aghilinejad et al., 2020; Ong et al., 2020) are available in the literature and provide additional information on flow patterns in aortic dissection which are not possible by imaging alone (Alimohammadi et al., 2015; Naim et al., 2016). While there are significant data supporting the impact of intimal septal motion on disease progression (Birjiniuk et al., 2017; Ong et al., 2020), past studies on dissection modeling assumed rigid vessel wall. This assumption leads to neglecting the septum dynamics and wall compliance which has been shown to play a critical role in understanding hemodynamics (Birjiniuk et al., 2017; Ong et al., 2020; Peelukhana et al., 2017). In addition, due to inability of such models in capturing wave dynamics, they are unable to describe detailed pulsatile flow and wave reflection (Rudenick et al., 2015; Ong et al., 2020). Our model is among the first which is able to capture the septal motion in TBAD. Results from simulations have been compared to invasively measured clinical data acquired during a TEVAR operation (Figure 5C) to verify the clinical relevancy of the computational model (Figure 6 and Table 2). The dimensionless pressure index inside the FL (RPP) was utilized to compare the *in vivo* results with our simulation. Table 2 shows that the calculated RPP from simulation data is within the range of clinical data and follow a similar trend. The computed flow waveform inside the FL (Figure 6B) shows the similar characteristics with the reported clinical MRI data in the literature (Rudenick et al., 2017). These confirm the physiological accuracy of our study for the purposes of investigating hemodynamics of TBAD.

### Impact of Endograft Length on LV Workload

The first novel finding in this study is related to examining the impact of endograft-aortic compliance mismatch on LV power requirement which is a global hemodynamic metric of the cardiovascular system. The replacement of highly elastic native aorta with non-compliant endograft reduces compliance and alters the aortic wave dynamics. This alteration has been shown to translate into additional workload on the LV, eventually inducing adaptive hypertrophy (Spadaccio et al., 2016). However, to the best of our knowledge, the effect of this compliance mismatch between the aorta and the endograft on hemodynamic variables has not been quantitatively studied. In this study, we investigated the effect of this compliance mismatch via changing the endograft length on LV pulsatile load. LV pulsatile load is the result of complex wave dynamics and LV-aorta coupling and has been used as a global hemodynamic index to monitor different wave conditions in the vasculature (Pahlevan and Gharib, 2013). Indeed, previous clinical studies suggested that reducing LV pulsatile load is an important therapeutic target in HF (Mitchell et al., 2001). Our results suggest a trend towards increased LV workload as endograft length increases at different heart rates (Figure 7). This finding is in line with previous observations in terms of increase in pulsatile load due to the overall decrease in aortic compliance (Pahlevan and Gharib, 2011a; Pahlevan and Gharib, 2013). While longer endografts have the advantage of covering more tears in AD, this undesirable effect can be a limiting factor for clinicians when choosing endograft length.

### Impact of Endograft Length on FL Thrombosis

Current understanding indicates that increased FL flow reversal enhances thrombosis, and patients exhibiting reversed flows within the FL may be more likely to develop complete FL thrombosis (Karmonik et al., 2012; Birjiniuk et al., 2017); this is considered as a positive prognostic indicator (Tsai et al., 2007; Thrumurthy et al., 2011). Figure 8 presents the spatial distributions of the flow velocity and wall displacement in the presence of short and long endografts. As expected, there is significant difference in the septum wall displacement during the cardiac cycle between these two models; the presence of a longer endograft leads to the decrease in the overall compliance of the system and smaller radial displacement of the intimal septum which is quantified in Figure 9. The velocity profile for short, medium, and long endografts is presented in Figure 10. Regarding the overall dynamics of the septum and the flow, lower compliance of the repaired aorta with longer endografts leads to the earlier development of the antegrade flow inside the false lumen. To be mentioned that RFI which is the measure for thrombose prediction is the ratio of the retrograde flow over the total flow. Therefore, although the amplitude of both the antegrade and retrograde component of the flow data is smaller inside in the model with longer endografts (Figure 10), the averaged RFI of different sites in these models is higher (Figure 11). In other words, our results suggest that increase in endograft length enhances FL flow reversal. This may be attributed to a reduction in the overall compliance of the septal wall as the native aorta is replaced with a rigid endograft, leading to less unidirectional flow into the FL and an increase in FL flow reversal. However, while the FL flow reversal enhances significantly as the endograft length increases from the short-size to medium-size (e.g., at HR=60 bpm , 65% increase in RFI from λ=0.13 to λ=0.40 ), there is a minor enhancement in RFI as the endograft length increases beyond λ=0.40 (e.g., at HR=60 bpm , a 12% increase in RFI from λ=0.40 to λ=0.66 ). This finding suggests that medium-size endograft replacement ( λ=0.40 ) may achieve high FL flow reversal (predictor of FL thrombosis) with minimal extra pulsatile load on LV.

### Effect of Medical Therapy on FL Thrombosis

Although many TBAD patients undergo surgical aortic repair, medical therapy remains an essential part of their treatment. The primary objective of this pharmacological therapy is the reduction of the rate of rise of systolic aortic pressure (Prêtre and Von Segesser, 1997; Mészáros et al., 2000; Baliga et al., 2007). Beta-blocking agents are the mainstay of pharmacologic therapy for TBAD as they reduce the HR and decrease the intrinsic contractile state of the heart. This study evaluated the effect of both these parameters (HR and LV contractility) on FL flow reversal. The results demonstrated that decreasing HR at a fixed CO enhances FL flow reversal (Figure 12B). Furthermore, lower HR led to increased flow reversal index (Figure 11B) after endograft deployment. This implies that lower HRs have favorable outcomes in terms of FL thrombus formation. To investigate the impact of different contractile states of LV on FL thrombosis, end-systolic elastance was decreased in our LV model to simulate the physiological response to beta blockers (reduced contractility). The results indicated that reduced contractility (at a fixed HR) enhances FL flow reversal. Ultimately, results suggest that medical therapy in TBAD patients not only achieves the therapeutic goal of reducing the systolic blood pressure (Figure 12A), but also contributes favorably to FL flow reversal (Figure 12B).

### Study Limitations

This study has certain limitations that should be considered. The dissection model used in this study is constructed based on average physiological values in TBAD patients and is based on a simplified (idealized) model of TBAD. This model is limited by the number of tears considered in the septum model as well as the exclusion of aortic branches and the aortic arch. While the geometry of TBAD can be very complex due to tortuosity, irregularities of luminal diameter along the dissection, multiple fenestrations in the septum wall and partial FL thrombosis, our model is intended to contribute to the understanding of the hemodynamics in TBAD independent of each individual. This generic model is ideal to provide insights on the impact of one parameter at a time (e.g., endograft length) while controlling all other parameters. We also utilized Newtonian flow assumption for the fluid in this study. This assumption is still conventionally used in both experimental and CFD studies in large arteries (Iskander et al., 2021). However, future studies are needed to investigate the significance of non-Newtonian flow behavior in TBAD modeling in terms of FL flow reversal after TEVAR. Another major assumption in this study is to model the endograft as a rigid material. While current commercially available endografts are not fully rigid, previous studies reported the measured elasticity of endografts are up to sixteen times larger than that of the aorta (Vardoulis et al., 2011). For this reason, the assumption of rigid endograft in this study is reasonable.

## Concluding Remarks

The present study provides a comprehensive analysis of the role of endograft length on both global and local hemodynamic variables in TBAD anatomy. The computational model used here illustrates the amplitude and the form of the septum displacement in TBAD (Figure 8, 9). The significance of the septum displacement necessitates the FSI modeling for capturing the wave dynamics in this disease. Trends towards increased FL flow reversal (Figure 11) and increased pulsatile workload with increasing the endograft lengths were observed (Figure 7). This trade-off between desirable impact on FL flow reversal via longer endografts and their undesirable impact on LV workload suggest that there may exist an optimal endograft length that can lead to improved long-term clinical outcomes. Based on the non-linear increase in FL flow reversal with increased endograft length (Figure 11), our results suggest medium-length endografts can lead to relatively high FL flow reversal (and consequent FL thrombosis) with minimal extra load on the LV. Another major finding of this study is related to the role of medical therapy on the hemodynamic state in TBAD. Our results indicate that medical therapy can achieve the therapeutic goal of reducing the systolic blood pressure and contribute favorably to FL flow reversal and FL thrombosis. Further clinical studies are needed to assess the role of endograft length on hemodynamic variables following TEVAR. Further patient-specific modeling can also be conducted utilizing the FSI approach to provide additional information on flow patterns and the comparison among different TBAD patients in the presence of the patient-specific septum dynamics. Employing such an approach is also helpful in identifying the possible factors involved in the formation of distal aneurysm and distal re-entry (Tse et al., 2011; Rudenick et al., 2015).

## Data Availability

The original contributions presented in the study are included in the article/supplementary material further inquiries can be directed to the corresponding author.

## References

[B1] Aghilinejad A. Amlani F. King K. S. Pahlevan N. M. (2020). Dynamic Effects of Aortic Arch Stiffening on Pulsatile Energy Transmission to Cerebral Vasculature as a Determinant of Brain-Heart Coupling. Sci. Rep. 10 (1), 8784–8812. 10.1038/s41598-020-65616-7 32472027 PMC7260194

[B2] Aghilinejad A. Amlani F. Liu J. Pahlevan N. M. (2021a). Accuracy and Applicability of Non-invasive Evaluation of Aortic Wave Intensity Using Only Pressure Waveforms in Humans. Physiol. Meas. 42 (10), 105003. 10.1088/1361-6579/ac2671 34521071

[B3] Aghilinejad A. Alavi R. Rogers B. Amlani F. Pahlevan N. M. (2021b). Effects of Vessel Wall Mechanics on Non-invasive Evaluation of Cardiovascular Intrinsic Frequencies. J. Biomechanics 129, 110852. 10.1016/j.jbiomech.2021.110852 34775340

[B4] Alimohammadi M. Sherwood J. M. Karimpour M. Agu O. Balabani S. Díaz-Zuccarini V. (2015). Aortic Dissection Simulation Models for Clinical Support: Fluid-Structure Interaction vs. Rigid Wall Models. Biomed. Eng. Online 14 (1), 34–16. 10.1186/s12938-015-0032-6 25881252 PMC4407424

[B5] Amlani F. Pahlevan N. M. (2020). A Stable High-Order FC-Based Methodology for Hemodynamic Wave Propagation. J. Comput. Phys. 405, 109130. 10.1016/j.jcp.2019.109130

[B6] Baliga R. Nienaber C. A. Isselbacher E. M. Eagle K. A. (2007). Aortic Dissection and Related Syndromes. Springer Science & Business Media, 260.

[B7] Berger D. S. Li J. K. Noordergraaf A. (1994). Differential Effects of Wave Reflections and Peripheral Resistance on Aortic Blood Pressure: a Model-Based Study. Am. J. Physiology-Heart Circulatory Physiology 266 (4), H1626–H1642. 10.1152/ajpheart.1994.266.4.h1626 8184943

[B8] Bilgi C. Atalık K. (2020). Effects of Blood Viscoelasticity on Pulsatile Hemodynamics in Arterial Aneurysms. J. Newt. Fluid Mech. 279, 104263. 10.1016/j.jnnfm.2020.104263

[B9] Birjiniuk J. Oshinski J. N. Ku D. N. Veeraswamy R. K. (2020). Endograft Exclusion of the False Lumen Restores Local Hemodynamics in a Model of Type B Aortic Dissection. J. Vasc. Surg. 71 (6), 2108–2118. 10.1016/j.jvs.2019.06.222 32446515

[B10] Birjiniuk J. Timmins L. H. Young M. Leshnower B. G. Oshinski J. N. Ku D. N. (2017). Pulsatile Flow Leads to Intimal Flap Motion and Flow Reversal in an *In Vitro* Model of Type B Aortic Dissection. Cardiovasc Eng. Tech. 8 (3), 378–389. 10.1007/s13239-017-0312-3 28608325

[B11] Birjiniuk J. Veeraswamy R. K. Oshinski J. N. Ku D. N. (2019). Intermediate Fenestrations Reduce Flow Reversal in a Silicone Model of Stanford Type B Aortic Dissection. J. biomechanics 93, 101–110. 10.1016/j.jbiomech.2019.06.019 31326118

[B12] Cheng Z. Wood N. B. Gibbs R. G. J. Xu X. Y. (2015). Geometric and Flow Features of Type B Aortic Dissection: Initial Findings and Comparison of Medically Treated and Stented Cases. Ann. Biomed. Eng. 43 (1), 177–189. 10.1007/s10439-014-1075-8 25092420

[B13] Collins J. S. Evangelista A. Nienaber C. A. Bossone E. Fang J. Cooper J. V. (2004). Differences in Clinical Presentation, Management, and Outcomes of Acute Type a Aortic Dissection in Patients with and without Previous Cardiac Surgery. Circulation 110 (11), II237237–42242. 10.1161/01.CIR.0000138219.67028.2a 15364869

[B14] Doyle J. F. (2001). Nonlinear Analysis of Thin-Walled Structures: Statics, Dynamics, and Stability. New York, NY: Springer Science & Business Media.

[B15] Engelmayr G. C. Jr Hildebrand D. K. Sutherland F. W. H. Mayer J. E. Jr Sacks M. S. (2003). A Novel Bioreactor for the Dynamic Flexural Stimulation of Tissue Engineered Heart Valve Biomaterials. Biomaterials 24 (14), 2523–2532. 10.1016/s0142-9612(03)00051-6 12695079

[B16] Girish A. Padala M. Kalra K. McIver B. V. Veeraswamy R. K. Chen E. P. (2016). The Impact of Intimal Tear Location and Partial False Lumen Thrombosis in Acute Type B Aortic Dissection. Ann. Thorac. Surg. 102 (6), 1925–1932. 10.1016/j.athoracsur.2016.05.020 27424468

[B17] Grinberg L. Karniadakis G. E. (2008). Outflow Boundary Conditions for Arterial Networks with Multiple Outlets. Ann. Biomed. Eng. 36 (9), 1496–1514. 10.1007/s10439-008-9527-7 18612828

[B18] Huang H. Lu X. Y. (2009). Theoretical and Numerical Study of Axisymmetric Lattice Boltzmann Models. Phys. Rev. E Stat. Nonlin Soft Matter Phys. 80 (1), 016701. 10.1103/PhysRevE.80.016701 19658832

[B19] Huang H. Wei H. Lu X.-Y. (2018). Coupling Performance of Tandem Flexible Inverted Flags in a Uniform Flow. J. Fluid Mech. 837, 461–476. 10.1017/jfm.2017.875

[B20] Huang W.-X. Shin S. J. Sung H. J. (2007). Simulation of Flexible Filaments in a Uniform Flow by the Immersed Boundary Method. J. Comput. Phys. 226 (2), 2206–2228. 10.1016/j.jcp.2007.07.002

[B21] Iskander A. Bilgi C. Naftalovich R. Hacihaliloglu I. Berkman T. Naftalovich D. (2021). The Rheology of the Carotid Sinus: A Path toward Bioinspired Intervention. Front. Bioeng. Biotechnol. 9, 439. 10.3389/fbioe.2021.678048 PMC8222608 34178967

[B22] Januzzi J. L. Isselbacher E. M. Fattori R. Cooper J. V. Smith D. E. Fang J. (2004). Characterizing the Young Patient with Aortic Dissection: Results from the International Registry of Aortic Dissection (IRAD). J. Am. Coll. Cardiol. 43 (4), 665–669. 10.1016/j.jacc.2003.08.054 14975480

[B23] Kang J. Aghilinejad A. Pahlevan N. M. (2019). On the Accuracy of Displacement-Based Wave Intensity Analysis: Effect of Vessel Wall Viscoelasticity and Nonlinearity. PloS one 14 (11), e0224390. 10.1371/journal.pone.0224390 31675382 PMC6824577

[B24] Karmonik C. Partovi S. Müller-Eschner M. Bismuth J. Davies M. G. Shah D. J. (2012). Longitudinal Computational Fluid Dynamics Study of Aneurysmal Dilatation in a Chronic DeBakey Type III Aortic Dissection. J. Vasc. Surg. 56 (1), 260–263. e261. 10.1016/j.jvs.2012.02.064 22579075

[B25] Karniadakis G. E. Karniadakis G. Sherwin S. (2005). Spectral/hp Element Methods for Computational Fluid Dynamics. Oxford University Press on Demand.

[B26] Karreman G. (1952). Some Contributions to the Mathematical Biology of Blood Circulation. Reflections of Pressure Waves in the Arterial System. Bull. Math. Biophysics 14 (4), 327–350. 10.1007/bf02477850

[B27] Kolh P. D'Orio V. Lambermont B. Gerard P. Gommes C. Limet R. (2000). Increased Aortic Compliance Maintains Left Ventricular Performance at Lower Energetic Cost. Eur. J. cardio-thoracic Surg. 17 (3), 272–278. 10.1016/s1010-7940(00)00341-9 10758388

[B28] Lee T. S. Huang H. Shu C. (2005). An Axisymmetric Incompressible Lattice BGK Model for Simulation of the Pulsatile Flow in a Circular Pipe. Int. J. Numer. Meth. Fluids 49 (1), 99–116. 10.1002/fld.997

[B29] Magee G. A. Veranyan N. Kuo E. C. Ham S. W. Ziegler K. R. Weaver F. A. (2019). Anatomic Suitability for "Off-The-Shelf" Thoracic Single Side-Branched Endograft in Patients with Type B Aortic Dissection. J. Vasc. Surg. 70 (6), 1776–1781. 10.1016/j.jvs.2019.04.461 31248760

[B30] Mathlouthi A. Nejim B. Magee G. A. Siracuse J. J. Malas M. B. (2021). Hospitalization Cost and In-Hospital Outcomes Following Type B Thoracic Aortic Dissection Repair. Ann. Vasc. Surg. 75, 22–28. 10.1016/j.avsg.2021.01.111 33819596

[B31] Mészáros I. Mórocz J. Szlávi J. Schmidt J. Tornóci L. Nagy L. (2000). Epidemiology and Clinicopathology of Aortic Dissection. Chest 117 (5), 1271–1278. 10.1378/chest.117.5.1271 10807810

[B32] Mitchell G. F. Tardif J.-C. Arnold J. M. O. Marchiori G. O’Brien T. X. Dunlap M. E. (2001). Pulsatile Hemodynamics in Congestive Heart Failure. Hypertension 38 (6), 1433–1439. 10.1161/hy1201.098298 11751731

[B33] Mittal R. Iaccarino G. (2005). Immersed Boundary Methods. Annu. Rev. Fluid Mech. 37, 239–261. 10.1146/annurev.fluid.37.061903.175743

[B34] Naim W. N. W. Ganesan P. B. Sun Z. Liew Y. M. Qian Y. Lee C.-J. (2016). Prediction of Thrombus Formation Using Vortical Structures Presentation in Stanford Type B Aortic Dissection: a Preliminary Study Using CFD Approach. Appl. Math. Model. 40 (4), 3115–3127. 10.1016/j.apm.2015.09.096

[B35] Olufsen M. S. (1999). Structured Tree Outflow Condition for Blood Flow in Larger Systemic Arteries. Am. J. physiology-Heart circulatory physiology 276 (1), H257–H268. 10.1152/ajpheart.1999.276.1.h257 9887040

[B36] Ong C. W. Wee I. Syn N. Ng S. Leo H. L. Richards A. M. (2020). Computational Fluid Dynamics Modeling of Hemodynamic Parameters in the Human Diseased Aorta: a Systematic Review. Ann. Vasc. Surg. 63, 336–381. 10.1016/j.avsg.2019.04.032 31344467

[B37] Pahlevan N. M. Amlani F. Hossein Gorji M. Hussain F. Gharib M. (2011). A Physiologically Relevant, Simple Outflow Boundary Model for Truncated Vasculature. Ann. Biomed. Eng. 39 (5), 1470–1481. 10.1007/s10439-011-0246-0 21240638

[B38] Pahlevan N. M. Gharib M. (2011a). Aortic Wave Dynamics and its Influence on Left Ventricular Workload. PloS one 6 (8), e23106. 10.1371/journal.pone.0023106 21853075 PMC3154923

[B39] Pahlevan N. M. Gharib M. (2011b). Low Pulse Pressure with High Pulsatile External Left Ventricular Power: Influence of Aortic Waves. J. biomechanics 44 (11), 2083–2089. 10.1016/j.jbiomech.2011.05.016 21679951

[B40] Pahlevan N. M. Gharib M. (2013). *In-vitro* Investigation of a Potential Wave Pumping Effect in Human Aorta. J. biomechanics 46 (13), 2122–2129. 10.1016/j.jbiomech.2013.07.006 23915578

[B41] Peelukhana S. V. Wang Y. Berwick Z. Kratzberg J. Krieger J. Roeder B. (2017). Role of Pulse Pressure and Geometry of Primary Entry Tear in Acute Type B Dissection Propagation. Ann. Biomed. Eng. 45 (3), 592–603. 10.1007/s10439-016-1705-4 27510916 PMC5331108

[B42] Peskin C. S. (2002). The Immersed Boundary Method. Acta Numer. 11, 479–517. 10.1017/s0962492902000077

[B43] Prêtre R. Von Segesser L. K. (1997). Aortic Dissection. Lancet 349 (9063), 1461–1464. 10.1016/S0140-6736(96)09372-5 9164331

[B44] Rudenick P. A. Bijnens B. H. García-Dorado D. Evangelista A. (2013). An *In Vitro* Phantom Study on the Influence of Tear Size and Configuration on the Hemodynamics of the Lumina in Chronic Type B Aortic Dissections. J. Vasc. Surg. 57 (2), 464e465–474. 10.1016/j.jvs.2012.07.008 23141674

[B45] Rudenick P. A. Bijnens B. H. Segers P. García-Dorado D. Evangelista A. (2015). Assessment of Wall Elasticity Variations on Intraluminal Haemodynamics in Descending Aortic Dissections Using a Lumped-Parameter Model. Plos one 10 (4), e0124011. 10.1371/journal.pone.0124011 25881158 PMC4399844

[B46] Rudenick P. A. Segers P. Pineda V. Cuellar H. García-Dorado D. Evangelista A. (2017). False Lumen Flow Patterns and Their Relation with Morphological and Biomechanical Characteristics of Chronic Aortic Dissections. Computational Model Compared with Magnetic Resonance Imaging MeasurementsComputational Model Compared with Magnetic Resonance Imaging Measurements. Plos One 12 (1), e0170888. 10.1371/journal.pone.0170888 28125720 PMC5270334

[B47] Spadaccio C. Nappi F. Al-Attar N. Sutherland F. W. Acar C. Nenna A. (2016). Old Myths, New Concerns: the Long-Term Effects of Ascending Aorta Replacement with Dacron Grafts. Not All that Glitters Is Gold. J. Cardiovasc. Trans. Res. 9 (4), 334–342. 10.1007/s12265-016-9699-8 PMC4990605 27245785

[B48] Tai N. R. Salacinski H. J. Edwards A. Hamilton G. Seifalian A. M. (2000). Compliance Properties of Conduits Used in Vascular Reconstruction. Br. J. Surg. 87 (11), 1516–1524. 10.1046/j.1365-2168.2000.01566.x 11091239

[B49] Takami Y. Tajima K. Kato W. Fujii K. Hibino M. Munakata H. (2012). Long-Term Size Follow-up of Knitted Dacron Grafts (Gelseal™) Used in the Ascending Aorta. Interact. Cardiovasc. Thorac. Surg. 14 (5), 529–531. 10.1093/icvts/ivr086 22345060 PMC3329298

[B50] Thrumurthy S. G. Karthikesalingam A. Patterson B. O. Holt P. J. E. Hinchliffe R. J. Loftus I. M. (2011). A Systematic Review of Mid-Term Outcomes of Thoracic Endovascular Repair (TEVAR) of Chronic Type B Aortic Dissection. Eur. J. Vasc. Endovascular Surg. 42 (5), 632–647. 10.1016/j.ejvs.2011.08.009 21880515

[B51] Tsai T. T. Evangelista A. Nienaber C. A. Myrmel T. Meinhardt G. Cooper J. V. (2007). Partial Thrombosis of the False Lumen in Patients with Acute Type B Aortic Dissection. N. Engl. J. Med. 357 (4), 349–359. 10.1056/nejmoa063232 17652650

[B52] Tse K. M. Chiu P. Lee H. P. Ho P. (2011). Investigation of Hemodynamics in the Development of Dissecting Aneurysm within Patient-Specific Dissecting Aneurismal Aortas Using Computational Fluid Dynamics (CFD) Simulations. J. biomechanics 44 (5), 827–836. 10.1016/j.jbiomech.2010.12.014 21256491

[B53] Vardoulis O. Coppens E. Martin B. Reymond P. Tozzi P. Stergiopulos N. (2011). Impact of Aortic Grafts on Arterial Pressure: a Computational Fluid Dynamics Study. Eur. J. Vasc. Endovascular Surg. 42 (5), 704–710. 10.1016/j.ejvs.2011.08.006 21889370

[B54] Vlachopoulos C. Aznaouridis K. Stefanadis C. (2010). Prediction of Cardiovascular Events and All-Cause Mortality with Arterial Stiffness: a Systematic Review and Meta-Analysis. J. Am. Coll. Cardiol. 55 (13), 1318–1327. 10.1016/j.jacc.2009.10.061 20338492

[B55] Wang Y. Shu C. Teo C. J. Wu J. (2015). An Immersed Boundary-Lattice Boltzmann Flux Solver and its Applications to Fluid-Structure Interaction Problems. J. Fluids Struct. 54, 440–465. 10.1016/j.jfluidstructs.2014.12.003

[B56] Williams D. M. LePage M. A. Lee D. Y. (1997). The Dissected Aorta: Part I. Early Anatomic Changes in an *In Vitro* Model. Radiology 203 (1), 23–31. 10.1148/radiology.203.1.9122399 9122399

[B57] Wu J. Shu C. (2009). Implicit Velocity Correction-Based Immersed Boundary-Lattice Boltzmann Method and its Applications. J. Comput. Phys. 228 (6), 1963–1979. 10.1016/j.jcp.2008.11.019

[B58] Yazdani A. Li H. Bersi M. R. Di Achille P. Insley J. Humphrey J. D. (2018). Data-driven Modeling of Hemodynamics and its Role on Thrombus Size and Shape in Aortic Dissections. Sci. Rep. 8 (1), 2515–2518. 10.1038/s41598-018-20603-x 29410467 PMC5802786

[B59] Yazdani A. Li H. Humphrey J. D. Karniadakis G. E. (2017). A General Shear-dependent Model for Thrombus Formation. PLoS Comput. Biol. 13 (1), e1005291. 10.1371/journal.pcbi.1005291 28095402 PMC5240924

[B60] Yin M. Ban E. Rego B. V. Zhang E. Cavinato C. Humphrey J. D. (2022). Simulating Progressive Intramural Damage Leading to Aortic Dissection Using DeepONet: an Operator–Regression Neural Network. J. R. Soc. Interface 19 (187), 20210670. 10.1098/rsif.2021.0670 35135299 PMC8826120

